# Adversity in childhood linked to elevated striatal dopamine function in adulthood

**DOI:** 10.1016/j.schres.2016.06.005

**Published:** 2016-10

**Authors:** Alice Egerton, Lucia R. Valmaggia, Oliver D. Howes, Fern Day, Christopher A. Chaddock, Paul Allen, Toby T. Winton-Brown, Michael A.P. Bloomfield, Sagnik Bhattacharyya, Jack Chilcott, Julia M. Lappin, Robin M. Murray, Philip McGuire

**Affiliations:** a King's College London, King's Health Partners, Institute of Psychiatry, Psychology and Neuroscience, De Crespigny Park, Denmark Hill, London SE5 8AF, UK; b Medical Research Council Clinical Sciences Centre, Institute of Clinical Sciences, Hammersmith Hospital, Imperial College London, Du Cane Road, London W12 0NN, UK

**Keywords:** Dopamine, Psychosis, Childhood adversity, PET, At risk mental state, social defeat

## Abstract

Childhood adversity increases the risk of psychosis in adulthood. Theoretical and animal models suggest that this effect may be mediated by increased striatal dopamine neurotransmission. The primary objective of this study was to examine the relationship between adversity in childhood and striatal dopamine function in early adulthood. Secondary objectives were to compare exposure to childhood adversity and striatal dopamine function in young people at ultra high risk (UHR) of psychosis and healthy volunteers. Sixty-seven young adults, comprising 47 individuals at UHR for psychosis and 20 healthy volunteers were recruited from the same geographic area and were matched for age, gender and substance use. Presynaptic dopamine function in the associative striatum was assessed using 18F-DOPA positron emission tomography. Childhood adversity was assessed using the Childhood Experience of Care and Abuse questionnaire. Within the sample as a whole, both severe physical or sexual abuse (T63 = 2.92; *P* = 0.005), and unstable family arrangements (T57 = 2.80; *P* = 0.007) in childhood were associated with elevated dopamine function in the associative striatum in adulthood. Comparison of the UHR and volunteer subgroups revealed similar incidence of childhood adverse experiences, and there was no significant group difference in dopamine function. This study provides evidence that childhood adversity is linked to elevated striatal dopamine function in adulthood.

## 1 Introduction

Traumatic experiences during childhood, such as physical, sexual or psychological abuse, increase the risk of mental illness in adulthood threefold (Varese et al., 2012). The neurobiological basis of this effect is unknown. However, exposure to sustained environmental stress elevates central dopaminergic neurotransmission in animal models (Antelman et al., 1980, Tidey and Miczek, 1996, Valenti et al., 2011), and an elevation in brain dopamine function is one of the most robust neurobiological features of psychosis (Howes et al., 2012). It has thus been suggested that psychosocial stress in childhood may increase the risk of psychosis in later life through an effect on dopaminergic neurotransmission (Howes et al., 2004, Selten et al., 2013, Thompson et al., 2004).

There is indirect evidence to support this notion from human neuroimaging studies. Healthy volunteers who have experienced childhood trauma show an elevated dopaminergic response to amphetamine administration, which may be mediated by perceived stress (Oswald et al., 2014). In addition, striatal dopamine release elicited by an acute psychosocial stress task is elevated in healthy college students who experienced low levels of maternal care during childhood (Pruessner et al., 2004), suggesting that childhood disadvantage may be associated with elevated dopaminergic responses to stress in later life. Compared to healthy volunteers, an elevated dopaminergic response to this stress task is also seen in adults who are at high risk for psychosis, adults with schizotypy, and patients with schizophrenia (Mizrahi et al., 2012, Soliman et al., 2008).

The social defeat hypothesis (Selten and Cantor-Graae, 2005, Selten et al., 2013) posits that repeated setbacks in social situations leads to a sensitization of mesostriatal dopamine neurotransmission, and thereby increases the risk of developing psychotic symptoms. Childhood trauma may represent one particular type of social defeat. In support of the social defeat hypothesis, it has recently been reported that amphetamine-stimulated dopamine release in the striatum is elevated in young adults with severe hearing impairment, who generally experience marked social exclusion (Gevonden et al., 2014).

The aim of the present study was to further examine how exposure to adversity in childhood impacts on striatal dopamine function in adulthood, in both a healthy volunteer group, and also a group at who are at ultra high risk (UHR) of developing psychosis. Both the level of striatal dopamine function and the incidence of childhood adversity tend to be higher in UHR groups, although to a lesser extent than in schizophrenia (Binbay et al., 2012, Egerton et al., 2013, Howes et al., 2009, Mizrahi et al., 2012). The primary hypothesis was that striatal presynaptic dopamine function would be elevated in both UHR and healthy volunteers who experienced childhood adversity. As previous studies have shown elevated dopamine responses in healthy volunteers who experienced childhood adversity (Oswald et al., 2014, Pruessner et al., 2004), we predicted that this relationship would be seen across both groups, but to a greater degree in the UHR sample. Related to this, the secondary hypotheses were that exposure to childhood adversity would be higher in the UHR than healthy volunteer group, and that striatal presynaptic dopamine function would be elevated in the UHR compared to the healthy volunteer group. A further hypothesis was that both striatal presynaptic dopamine and childhood adversity would be positively related to the severity of psychotic symptoms.

## 2 Participants and methods

### 2.1 Participants

This study had National Health Service Research Ethics Committee and Administration of Radioactive Substances Advisory Committee approval. All participants provided their written informed consent. One group consisted of 47 individuals who met operationalized UHR criteria (over the last year, experience of attenuated psychotic symptoms, or psychotic symptoms that lasted less than a week and spontaneously remitted, or schizotypal personality disorder or a first degree relative with a psychotic disorder plus a decline in functioning) (Phillips et al., 2000) recruited from Outreach and Support in South London (OASIS), part of the South London and Maudsley National Health Service Trust. A second group comprised 20 healthy volunteers (Control), recruited from the same geographic area by public advertisement. They had no personal or family history of psychiatric symptoms, and were not taking psychotropic medication. Both groups included subjects who had participated in previous dopamine imaging studies (Egerton et al., 2013, Howes et al., 2011a, Howes et al., 2009), and represent those participants in whom there was existing cross-over with a separate study on childhood adversity, or those whom could be re-contacted to complete the childhood adversity questionnaire.

### 2.2 Assessment of clinical variables and childhood adversity

Psychopathology was assessed using the Comprehensive Assessment of At-Risk Mental States (CAARMS) (Phillips et al., 2000), Positive and Negative Syndrome Scale for Schizophrenia (PANSS) (Kay et al., 1987), and the Hamilton Depression and Anxiety Rating Scales (Hamilton, 1959, Hamilton, 1960).

Childhood adversity was assessed using the Childhood Experience of Care and Abuse Questionnaire (CECA-Q) (Bifulco et al., 2005). Participants did not have to answer any questions they were uncomfortable with. The following adverse events were analysed: A) death or separation from either parental figure; B) severe sexual or physical abuse; C) maternal or paternal antipathy or neglect; D) more than two family arrangements (the number of different caregivers with each of whom the child lived for at least one year). Each was rated as either present (exposure) or absent (no exposure). As only four participants reported being brought up in a children's home or institution, this event was excluded from the analysis.

### 2.3 18F-DOPA PET imaging

All subjects were studied using 18F-DOPA positron emission tomography (PET) imaging. Data were acquired on either a CTI/Siemens ECAT HR + 962 tomograph (11 healthy volunteers; 28 UHR) or a CTI/Siemens ECAT HR ++ 966 tomograph (9 healthy volunteers; 19 UHR) (Siemens Molecular Imaging, Knoxville, TN, U.S.A.) as previously described (Egerton et al., 2013, Howes et al., 2009). Study participants were asked to fast for 12 h before imaging. Urine drug screens on the morning of the scan to confirmed absence of illicit substance use. All subjects received carbidopa (150 mg) and entacapone (400 mg) orally 1 h before imaging to reduce the formation of radiolabeled 18F-DOPA metabolites. All data were acquired in three-dimensional mode. A 10-minute (ECAT HR + 962) or 5-minute (ECAT HR + 966) transmission scan was performed before radiotracer injection to correct for attenuation and scatter. Thirty seconds after the start of PET image acquisition, 180 Mbq (ECAT HR + 962) or 150 MBq (ECAT HR + 966) 18F-DOPA (± 10%) was administered by bolus intravenous injection. On both scanners, emission data were acquired in list mode for 95 min, and rebinned into 26 time frames.

Data were reconstructed using the 3D reprojection algorithms. Head movement was corrected for by realigning denoised (Turkheimer et al., 1999), nonattenuation-corrected dynamic images (Studholme et al., 1996) and applying the transformation parameters to the corresponding attenuation-corrected frames to create a movement-corrected dynamic image for analysis. Standardized striatal volumes of interest (VOI) were delineated bilaterally on a single subject T1 magnetic resonance image in Montreal Neurologic Institute (MNI) space. These VOI included the limbic (ventral), associative and sensorimotor subdivisions of the striatum, as according to previously defined anatomical criteria (Martinez et al., 2003, Mawlawi et al., 2001). The cerebellar reference region was defined using a probabilistic atlas (Hammers et al., 2003). An 18F-DOPA template, also in MNI space, was then normalized together with the VOI map to each individual PET summation image using SPM5 (http://fil.ion.ucl.ac.uk/spm; Wellcome Department of Imaging Neuroscience, University College London). Graphical analysis, adapted for a reference tissue input function (Patlak and Blasberg, 1985, Turkheimer et al., 2006) as used to estimate presynaptic dopamine synthesis capacity by calculating the rate of utilization of the dopamine precursor ^18^F-DOPA in the bilateral striatum, relative to the cerebellar reference tissue (*k*_i_^cer^ min^− 1^). To control for effects of scanner model (Egerton et al., 2013), individual subject *k*_i_^cer^ values were converted to *z*-scores (z = (*k*_i_^cer^ – scanner mean *k*_i_^cer^)/scanner standard deviation (SD)) for all analyses.

### 2.4 Statistical analyses

Statistical analysis was performed in SPSS version 19.0 (IBM Corporation, Armonk, New York). For demographic, clinical variables and measures of childhood adversity, comparisons between Control and UHR groups were performed using Fisher's Exact, Mann-Whitney *U* or unpaired *t*-tests. The threshold for statistical significance of *P* < 0.05. The same approach was used to compare demographic and clinical variables in individuals exposed or not exposed to each type of childhood adversity. The impact of exposures to childhood adversity on striatal dopamine function were determined using independent samples *t*-tests (exposure versus non-exposure). Effect size was calculated as Cohen's d. Secondary analysis employed univariate ANOVA to explore potential influences of group (Control versus UHR) and substance use on the relationships between exposures to childhood adversity and dopamine function. After confirming normality of distribution, relationships between the severity of total prodromal symptoms (total CAARMS score) in the UHR group and striatal dopamine function (*z*-score) were explored Pearson's correlation coefficient.

## 3 Results

### 3.1 Description of the UHR and control samples

There were no significant differences between the UHR and Control groups in demographic features or substance use (Table 1). As expected, all symptom scores were significantly higher in the UHR group (Table 1). Two of the UHR participants were taking antipsychotic medication (olanzapine, 7.5 mg; quetiapine, 25 mg).

### 3.2 Experiences of childhood adversity

Within the total sample, the proportion of participants reporting each type of childhood adversity ranged from 25% for more than two family arrangements, to 62% for parental loss or separation (Table 2). Within the total sample, there were no differences in demographic measures or reported substance misuse between participants who reported exposure to each type of childhood adversity and participants who did not (Supplementary Table S1).

### 3.3 Presynaptic dopamine function following childhood adversity

**Fig. 1:**
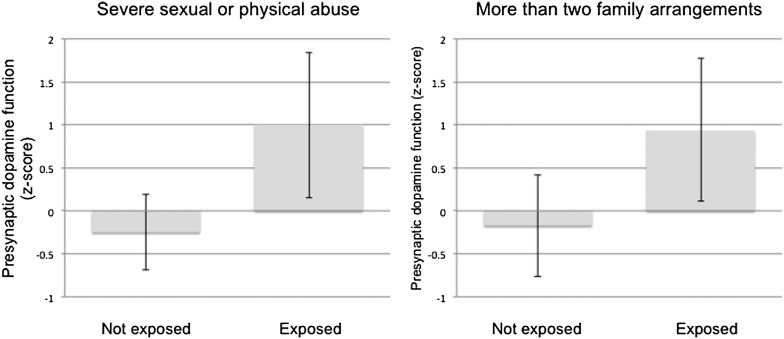
Presynaptic dopamine function (18F-DOPA *k*_i_^cer^*z*-score) in the associative striatum is elevated in young adults who experienced severe sexual or physical abuse (left panel) or unstable family arrangements (right panel) during childhood.

In the associative striatum, dopamine function was significantly higher in participants who had experienced severe physical or sexual abuse in childhood compared to those who had not (T63 = 2.92; *P* = 0.005; Table 3; Fig. 1, left). Dopamine function in this region was also elevated in participants who had experienced more than two family arrangements compared to those who had not (T57 = 2.80; *P* = 0.007; Table 3; Fig. 1, right). Both of these findings were associated with medium to large effect sizes (Cohen's d = 0.75 and 0.86 respectively).

Overall, of the twenty-six participants who reported sexual or physical abuse, eight also reported multiple family arrangements. When both physical/sexual abuse and multiple family arrangements were included in the same model, the effect of multiple family arrangements remained significant (*F*1,58 = 5.96; *P* = 0.018), while the effect of sexual/physical abuse approached significance (*F*1,58 = 3.50; *P* = 0.067). The elevations in dopamine function remained significant when the two UHR individuals who were taking antipsychotic medication were excluded from the analysis (sexual or physical abuse: T61 = 2.75; *P* = 0.008; family arrangements: T55 = 2.85; *P* = 0.005), and when group (UHR or Control) was included as a factor in the analyses (sexual or physical abuse: *F*1,61 = 6.53; *P* = 0.01; more than two family arrangements: *F*1,55 = 0.50; *P* = 0.02). There were no significant interactions between group and adversity (sexual or physical abuse * group interaction: *F*1,61 = 0.29; *P* = 0.59; more than two family arrangements * group interaction: *F*1,55 = 0.27; *P* = 0.60). When analysis was restricted to only the UHR group, these elevations approached significance (both *P* = 0.06; Supplementary Table S2). The elevations in associative striatal dopamine function across UHR and Control participants exposed to either sexual or physical abuse, or to more than two family arrangements, also remained significant when age, gender, smoking or alcohol drinking status, or use of cannabis, cocaine, amphetamine, ecstasy or ketamine were entered as fixed factors in the analyses (all *P* < 0.05). The same pattern of findings was evident when the region of interest comprised the whole striatum, with both severe physical or sexual abuse and multiple family arrangements again associated with significantly elevated dopamine function (Supplementary Table S3), albeit with slightly smaller effect sizes than for the associative striatum (d = 0.55 and 0.77 respectively).

Experiencing more than two family arrangements was also associated with increased dopamine function in the sensorimotor striatum (T57 = 2.40; *P* = 0.02; d = 0.76; Supplementary Table S3), but otherwise dopamine function in the limbic and sensorimotor subregions was not significantly different in participants who did and did not report adverse childhood experiences.

### 3.4 Childhood adversity in the UHR compared to control group

When exposures to each type of childhood adversity in UHR and volunteer groups were compared, only physical or sexual abuse was more commonly reported in the UHR group (*P* = 0.03; Table 2).

### 3.5 Presynaptic dopamine function in the UHR compared to Control group

There were no significant differences in striatal presynaptic dopamine function between the UHR and Control groups (Table 4).

### 3.6 Relationships between dopamine function or childhood adversity and symptoms in the UHR group

In the UHR group, the positive correlation between the severity of total prodromal symptoms (total CAARMS score) and dopamine function (*z*-score) was significant in the sensorimotor striatum (n = 47; r = 0.30; *P* = 0.04) but not in the associative (n = 47; r = 0.20; *P* = 0.19), limbic (n = 47; r = 0.04; *P* = 0.80 or whole striatum (n = 47; r = 0.23; *P* = 0.13). There were no significant differences in prodromal symptom severity, anxiety or depression between individuals who had or had not been exposed to each type of adversity (*P* > 0.05).

## 4 Discussion

An elevation in striatal dopamine function in adults who have been exposed to childhood adversity is consistent with animal studies linking psychogenic stress to striatal dopaminergic elevation (Antelman et al., 1980, Tidey and Miczek, 1996, Valenti et al., 2011), and with the recent finding of elevated dopaminergic responses to amphetamine in healthy volunteers exposed to childhood adversity (Oswald et al., 2014). Elevated dopamine function in the associative striatum was linked to severe (sexual or physical) abuse, and to multiple family arrangements, which may be a proxy marker for other stressors, including abuse (Casanueva et al., 2014). Exposure to stressors in childhood and elevated brain dopamine function in early adulthood have each been independently identified as major psychosocial and neurobiological risk factors for psychosis (Howes et al., 2011b, Howes et al., 2012, Varese et al., 2012). Our findings link these factors in the same individuals, and illustrate how adverse environmental influences could impact on brain development to alter the risk of a later psychotic illness (Howes et al., 2004, Selten et al., 2013, Thompson et al., 2004).

Physical and sexual abuse can be viewed as a forms of intentional harm which lead to enduring feelings of humiliation, which would place our findings of increased striatal dopamine function in people who experienced these forms of abuse as consistent with the social defeat hypothesis (Selten et al., 2013). In relation to this, it is perhaps interesting that we found no association between dopamine function and parental loss or separation, which are forms of trauma that do not necessarily or directly involve intentional harm to the child (Selten et al., 2013).

The elevation in striatal dopamine function in subjects with a history of childhood adversity was not attributable to increased substance use in these individuals, as they did not use illicit drugs more than individuals who had not experienced adverse events in childhood, and the findings remained significant after controlling for substance misuse. The relationships between childhood adverse events and dopamine function were not corrected for multiple comparisons. While the reported relationships between multiple family arrangements or physical or sexual abuse and dopamine function in the associative striatum would have survived correction, the relationship between multiple family arrangements and dopamine in the sensorimotor striatum would not, and therefore warrants confirmation in a larger sample.

There was no evidence that the relationship between childhood adversity and dopamine function was different in people with a high risk of psychosis and healthy volunteers, although the relatively small number of participants in the healthy volunteer group may have limited the power to observe a difference in the relationship between adversity and dopamine function between these groups. While non-significant, the tendency towards higher levels of both striatal dopamine function and of exposure to adversity in the UHR group may suggest that the significant relationships between adversity and elevated dopamine function across UHR and healthy volunteers were primarily driven by the UHR group, and when analysis was restricted to the UHR group alone these findings approached significance. The elevation in dopamine function in those exposed to childhood adversity was particularly evident in the associative subdivision of the striatum. This is the same striatal subregion where the most robust dopaminergic findings are seen in schizophrenia (Howes and Kapur, 2009, Kegeles et al., 2010).

The reported incidence of childhood adversities by both the UHR and Control participants was relatively high. The volunteers were recruited from a geographical area (in South East London) that has unusually high levels of social and economic deprivation, and previous studies have found that childhood adversity is relatively common among the local population (Frissa et al., 2013). However meta-analysis in patients with psychotic disorders suggest that they are nearly three times more likely to have experienced childhood adversities than controls (Varese et al., 2012).

In the individuals included in the present study, striatal dopamine function was numerically but not significantly elevated in the UHR compared to healthy volunteer group. This cohort represents a subsample from our previous studies that detected a significant elevation in striatal dopamine function in UHR compared to healthy volunteers overall (Egerton et al., 2013, Howes et al., 2009). In UHR samples, the elevation in dopamine function is less marked than in schizophrenia (Howes et al., 2009, Mizrahi et al., 2012) and is mainly driven by the subgroup of UHR subjects who later develop psychosis (Howes et al., 2011b). This, along with the relatively high incidences of childhood adversities in the healthy volunteer sample, may account for the overlap in values for striatal dopamine synthesis capacity between the UHR and healthy volunteers.

The putative relationship between childhood adversity, dopamine function and risk of psychosis could be further investigated in a large prospective study, which would determine how these factors relate to long-term clinical outcome in UHR subjects. However, an effect of adverse psychosocial experiences on dopamine function might only impact on the risk of illness if there are interactions with other factors that have also been implicated in psychosis, such as psychosocial exposures in adulthood (Morgan et al., 2014) or genetic risk factors (Modinos et al., 2013). This would be consistent with current aetiological models of mental illness, which propose that these involve complex gene-environment, gene-gene and environment-environment interactions (van Winkel et al., 2013).

One limitation of the present study (and most other adult studies of childhood abuse) is that the assessment of childhood adversity relied on retrospective reporting, which may be influenced by current psychopathology (Lysaker et al., 2005, Wolkind and Coleman, 1983). Nonetheless patients' retrospective reports of abuse tend to be stable, accurate and unaffected by current symptoms (Fisher et al., 2011). The low number of exposures to some forms or markers of psychosocial stress (such as being taken into local authority care) limited the analysis to relatively common factors. These issues could be addressed in large scale prospective studies of children with detailed psychosocial evaluations, neuroimaging measures, and long term follow up.

## Disclosure

AE has received consultancy fees from Heptares Therapeutics Ltd. and worked on research funded by Hoffman la Roche. ODH has received unrestricted investigator-led charitable funding from or spoken at meetings organized by Astra-Zeneca, Bristol-Myers Squibb, Janssen, Hoffman la Roche, Leyden-Delta and Eli Lilly. RMM has received honoraria from Janssen, Eli Lilly, Astra-Zeneca, Bristol-Myers Squibb, and Hoffman la Roche. PM has received consultancy fees from Hoffman la Roche and Sunovion. The remaining authors declare no potential conflicts of interest.

## Conflict of interest

AE has received consultancy fees from Heptares Therapeutics Ltd. and worked on research funded by Hoffman la Roche. ODH has received unrestricted investigator-led charitable funding from or spoken at meetings organized by Astra-Zeneca, Bristol-Myers Squibb, Janssen, Hoffman la Roche, Leyden-Delta and Eli Lilly. RMM has received honoraria from Janssen, Eli Lilly, Astra-Zeneca, Bristol-Myers Squibb, and Hoffman la Roche. PM has received consultancy fees from Hoffman la Roche and Sunovion. The remaining authors declare no potential conflicts of interest.

## Contributors

AE, LV, OH, PA, RM and PM designed the study. AE, LV, OH, FD, CC, TW-B, MB, SB acquired the study data; AE, FD, MB, JC, JL analysed the study data. All authors contributed to and approved the final manuscript.

## Role of funding source

The funding source had no role in the design and conduct of the study; collection, management, analysis, and interpretation of the data; preparation, review, or approval of the manuscript; and decision to submit the manuscript for publication.

## Figures and Tables

**Table 1:** Demographic characteristics, substance use and symptoms in the total sample, control and UHR groups.

	Total (n = 67)	Control (n = 20)	UHR (n = 47)	Statistic
Demographics				
Male/female	39/28	12/8	27/20	*P* > 0.99
Age, mean ± sd	23.7 ± 4.5	23.8 ± 4.3	23.6 ± 4.6	T = 0.15; *P* = 0.88
Handedness, right/left	58/9	16/4	42/5	*P* = 0.43
Substance use				
Non-smoker/smoker	35/22	13/7	22/25	*P* = 0.19
Non-drinker/drinker	17/50	4/16	13/34	*P* = 0.55
Cigarettes/day	3.8 ± 5.3	2.3 ± 3.8	4.5 ± 5.7	T = 1.58; *P* = 0.12
Alcohol units/week	6.7 ± 7.9	8.4 ± 8.6	6.0 ± 7.6	T = 0.26; *P* = 0.26
Cannabis 0/1/2/3/4	20/18/9/9/11	8/6/3/0/3	12/12/6/9/8	*P* > 0.99
Cocaine 0/1/2/3/4	43/14/4/4/2	16/3/0/1/0	27/11/4/3/2	*P* > 0.99
Amphetamine 0/1/2/3/4	52/11/1/3/0	18/2/0/0/0	34/9/1/3/0	*P* > 0.99
Ecstasy 0/1/2/3/4	39/18/6/4/0	14/3/3/0/0	25/15/3/4/0	*P* > 0.99
Ketamine 0/1/2/3/4	61/3/1/1/0	18/1/0/0/0	43/2/1/1/0	*P* > 0.99
Symptoms				
CAARMS total		2.2 ± 2.8	41.3 ± 18.1	T = 9.59; *P* < 0.001
CAARMS positive		0.3 ± 0.6	7.7 ± 3.3	T = 10.0; *P* < 0.001
PANSS positive		7.0 ± 0.0	12.9 ± 4.3	T = 6.10; *P* < 0.001
PANSS negative		7.0 ± 0.0	11.5 ± 5.2	T = 3.90; *P* < 0.001
PANSS general		16.3 ± 0.6	27.2 ± 7.6	T = 6.44; *P* < 0.001
PANSS total		30.3 ± 0.6	51.7 ± 14.3	T = 6.68; *P* < 0.001
Hamilton anxiety		0.8 ± 1.2	11.9 ± 9.8	T = 5.03; *P* < 0.001
Hamilton depression		0.8 ± 1.2	11.6 ± 8.4	T = 5.77; *P* < 0.001

CAARMS: Comprehensive Assessment for At Risk Mental States; PANSS: Positive And Negative Syndrome Scale. For cannabis, cocaine, amphetamine, ecstasy and ketamine use, categories of 0/1/2/3/4 indicate never used/very occasional or experimental use/occasional or monthly use/moderate or weekly use/severe or daily use respectively.

**Table 2:** Exposure to childhood adversity in the total sample, control and UHR groups.

	Total	Control	UHR	*P* value
Parental loss or separation	40/65 (62%)	10/20 (50%)	30/45 (67%)	0.27
Severe physical or sexual abuse	26/65 (40%)	4/20 (20%)	22/45 (49%)	0.03
Severe antipathy or neglect	21/59 (36%)	7/20 (35%)	14/39 (36%)	> 0.99
More than two family arrangements	15/59 (25%)	3/20 (15%)	12/39 (31%)	0.22

Data are presented as the number and percentage of participants reporting exposure/group total.

**Table 3:** Associative Striatal Dopamine Function and Childhood Adversity.

	No exposure	Exposure	Statistic, effect size
Parental loss or separation	0.11 ± 0.71	− 0.02 ± 1.14	T63 = 0.53; *P* = 0.60; d = 0.14
Severe physical or sexual abuse	− 0.25 ± 1.00	0.44 ± 0.84	**T63** **=** **2.92; *P*** **=** **0.005; d** **=** **0.75**
Severe antipathy or neglect	0.09 ± 0.94	− 0.09 ± 1.03	T57 = 0.68; *P* = 0.50; d = 0.18
More than two family arrangements	− 0.17 ± 0.94	0.59 ± 0.83	**T57** **=** **2.80; *P*** **=** **0.007; d** **=** **0.86**

Dopamine function is expressed as the mean ± standard deviation *z*-scores for 18F-DOPA *k*_i_^cer^ values, representing presynaptic dopamine synthesis capacity.

Statistics in bold type indicate significant results (*P* < 0.05).

**Table 4:** Striatal dopamine function in the control and UHR groups.

	Control (n = 20)	UHR (n = 47)	Statistic
Whole striatum	0.30 ± 1.01	0.54 ± 1.08	*T* = 0.88; *P* = 0.38
Associative striatum	− 0.20 ± 0.92	0.09 ± 1.02	*T* = 1.08; *P* = 0.28
Sensorimotor striatum	0.80 ± 1.24	1.10 ± 1.49	*T* = 0.78; *P* = 0.44
Limbic striatum	0.43 ± 1.99	0.55 ± 1.23	*T* = 0.31; *P* = 0.76

Dopamine function is expressed as the mean ± standard deviation *z*-scores for 18F-DOPA *k*_i_^cer^ values, representing presynaptic dopamine synthesis capacity.

## Appendix A Supplementary data

Supplementary tables. Image 1
