# Supplementary material for: Adversity in childhood linked to elevated striatal dopamine function in adulthood
Source: Schizophr Res. 2016 Oct;176(2-3):171–6. doi: 10.1016/j.schres.2016.06.005 (PMC5147458; doi:10.1016/j.schres.2016.06.005)
Supplement: Supplementary file 1 — Supplementary tables. [file mmc1.docx]

**Supplementary Table S1. Demographics and Substance Use by Exposure to Childhood Adversity**

|  | | **No Exposure** | **Exposure** | **Statistic** |
| --- | --- | --- | --- | --- |
| **Parental loss or separation** | | | | |
|  | Male / female | 16/9 | 22/18 | P = 0.61 |
|  | Age, mean ± sd | 23.2 ± 3.9 | 23.7 ± 4.7 | T = 0.41; P = 0.68 |
|  | Handedness, right/left | 22/3 | 34/6 | P > 0.99 |
|  | Non-smoker/smoker | 17/8 | 18/22 | P = 0.08 |
|  | Non-drinker /drinker | 8/17 | 9/31 | P = 0.56 |
|  | Cigarettes / day | 2.48 ± 4.01 | 4.50 ± 5.91 | T63 = 1.50; P = 0.14 |
|  | Alcohol units / week | 7.40 ± 7.90 | 5.95 ± 8.03 | T63 = 0.71; P = 0.48 |
|  | Cannabis 0/1/2/3/4 | 8/6/4/1/6 | 12/12/5/7/4 | P > 0.99 |
|  | Cocaine 0/1/2/3/4 | 14/7/1/2/1 | 28/7/2/2/1 | P > 0.99 |
|  | Amphetamines 0/1/2/3/4 | 17/6/0/2/0 | 34/4/1/1/0 | P > 0.99 |
|  | Ecstasy 0/1/2/3/4 | 11/9/4/1/0 | 28/7/2/3/0 | P > 0.99 |
|  | Ketamine 0/1/2/3/4 | 22/2/0/0/0 | 37/1/1/1/0 | P > 0.99 |
| **Severe physical or sexual abuse** | | | | |
|  | Male / female | 24/15 | 14/12 | P = 0.61 |
|  | Age, mean ± sd | 23.41 ± 4.51 | 23.69 + 4.23 | T = 0.25; P = 0.80 |
|  | Handedness, right/left | 33/6 | 23/3 | P = 0.73 |
|  | Non-smoker/smoker | 24/15 | 11/15 | P = 0.20 |
|  | Non-drinker /drinker | 12/27 | 5/21 | P = 0.39 |
|  | Cigarettes / day | 3.10 ± 5.21 | 4.65 ± 5.44 | T63 = 1.16; P = 0.25 |
|  | Alcohol units / week | 5.47 ± 6.89 | 8.07 ± 9.23 | T63 = 1.30; P = 0.20 |
|  | Cannabis 0/1/2/3/4 | 14/12/4/5/4 | 6/6/5/3/6 | P > 0.99 |
|  | Cocaine 0/1/2/3/4 | 27/8/2/1/1 | 15/6/1/3/1 | P > 0.99 |
|  | Amphetamines 0/1/2/3/4 | 31/6/0/2/0 | 20/4/1/1/0 | P > 0.99 |
|  | Ecstasy 0/1/2/3/4 | 24/9/4/2/0 | 15/7/2/2/0 | P > 0.99 |
|  | Ketamine 0/1/2/3/4 | 35/2/1/0/0 | 24/1/0/1/0 | P > 0.99 |
| **Severe antipathy or neglect** | | | | |
|  | Male / female | 22/16 | 12/9 | P > 0.99 |
|  | Age, mean ± sd | 22.7 ± 4.64 | 24.6 ± 4.04 | T57 = 1.52; P = 0.13 |
|  | Handedness, right/left | 6/32 | 2/19 | P = 0.70 |
|  | Non-smoker/smoker | 25/13 | 9/12 | P = 0.11 |
|  | Non-drinker /drinker | 12/26 | 3/18 | P = 0.21 |
|  | Cigarettes / day | 2.42 ± 4.03 | 4.48 ± 6.38 | T57 = 1.51; P = 0.14 |
|  | Alcohol units / week | 6.61 ± 8.00 | 6.96 ± 8.37 | T57 = 0.16; P = 0.87 |
|  | Cannabis 0/1/2/3/4 | 13/9/5/5/6 | 7/7/3/2/2 | P > 0.99 |
|  | Cocaine 0/1/2/3/4 | 24/10/2/2/0 | 16/3/1/1/0 | P > 0.99 |
|  | Amphetamines 0/1/2/3/4 | 32/5/0/1/0 | 15/4/1/1/0 | P > 0.99 |
|  | Ecstasy 0/1/2/3/4 | 21/10/5/2/0 | 14/4/1/2/0 | P > 0.99 |
|  | Ketamine 0/1/2/3/4 | 35/3/0/0/0 | 18/0/1/1/0 | P > 0.99 |
|  |  | **No Exposure** | **Exposure** | **Statistic** |
| **More than two family arrangements** | | | | |
|  | Male / female | 25/19 | 9/6 | P > 0.99 |
|  | Age, mean ± sd | 23.11 ± 3.89 | 24.20 ± 6.01 | T57 = 0.81; P = 0.42 |
|  | Handedness, right/left | 39/5 | 12/3 | P = 0.67 |
|  | Non-smoker/smoker | 26/18 | 8/7 | P = 0.77 |
|  | Non-drinker /drinker | 12/32 | 3/12 | P = 0.74 |
|  | Cigarettes / day | 3.16 ± 4.91 | 3.13 ± 5.58 | T57 = 0.12; P = 0.99 |
|  | Alcohol units / week | 6.99 ± 8.42 | 5.97 ± 7.13 | T57 = 0.42; P = 0.67 |
|  | Cannabis 0/1/2/3/4 | 16/12/5/4/7 | 4/4/3/3/1 | P > 0.99 |
|  | Cocaine 0/1/2/3/4 | 31/7/3/3/0 | 9/6/0/0/0 | P > 0.99 |
|  | Amphetamines 0/1/2/3/4 | 35/6/1/2/0 | 12/3/0/0/0 | P > 0.99 |
|  | Ecstasy 0/1/2/3/4 | 25/9/6/4/0 | 10/5/0/0/0 | P > 0.99 |
|  | Ketamine 0/1/2/3/4 | 38/3/1/1/0 | 15/0/0/0/0 | P > 0.99 |

For cannabis, cocaine, amphetamine, ecstasy and ketamine use, categories of 0/1/2/3/4 indicate never used / very occasional or experimental use / occasional or monthly use / moderate or weekly use / severe or daily use respectively.

**Supplementary Table S2. Associative Striatal Dopamine Function and Childhood Adversity in the UHR group**

|  | No exposure | Exposure | Statistic, Effect Size |
| --- | --- | --- | --- |
| Parental loss or separation | 0.31 ± 0.67 | 0.04 ± 1.15 | T43 = 0.83; P = 0.41; d = 0.29 |
| Severe physical or sexual abuse | -0.15 ± 1.08 | 0.42 ± 0.88 | T43 = 1.93; P = 0.06; d = 0.58 |
| Severe antipathy or neglect | 0.22 ± 0.86 | -0.01 ± 1.19 | T37 = 0.68; P = 0.50; d = 0.22 |
| More than two family arrangements | -0.06 ± 0.98 | 0.58 ± 0.86 | T37 = 1.95; P = 0.06; d = 0.69 |

Dopamine function is expressed as the mean ± standard deviation z-scores for 18F-DOPA k_i_^cer^ values, representing presynaptic dopamine synthesis capacity.

**Supplementary Table S3. Dopamine function in the Whole, Sensorimotor and Limbic Striatum by Exposure to Childhood Adversity**

|  | **No exposure** | **Exposure** | **Statistic** |
| --- | --- | --- | --- |
| **Parental loss or separation** | | | |
| Whole striatum | 0.65 ± 0.73 | 0.42 ± 1.19 | T63 = 0.86; P = 0.39 |
| Sensorimotor striatum | 1.27 ± 1.24 | 0.96 ± 1.47 | T63 = 0.88; P = 0.38 |
| Limbic striatum | 0.89 ± 0.88 | 0.34 ± 1.72 | T63 = 1.49; P = 0.14 |
| **Severe sexual or physical abuse** | | | |
| Whole striatum | 0.29 ± 1.07 | 0.84 ± 0.91 | T63 = 2.12; P = 0.04* |
| Sensorimotor striatum | 0.84 ± 1.44 | 1.43 ± 1.25 | T63 = 1.72; P = 0.09 |
| Limbic striatum | 0.70 ± 1.19 | 0.33 ± 1.81 | T63 = 0.99; P = 0.32 |
| **Severe antipathy or neglect** | | | |
| Whole striatum | 0.63 ± 0.99 | 0.29 ± 1.09 | T57 = 1.20; P = 0.23 |
| Sensorimotor striatum | 1.12 ± 1.21 | 1.02 ± 1.67 | T57 = 0.25; P = 0.80 |
| Limbic striatum | 0.77 ± 1.69 | 0.17 ± 1.05 | T57 = 1.47; P = 0.15 |
| **More than two family arrangements** | | | |
| Whole striatum | 0.32 ± 1.01 | 1.06 ± 0.90 | T57 = 2.50; P = 0.02* |
| Sensorimotor striatum | 0.84 ± 1.37 | 1.80 ± 1.16 | T57 = 2.40; P = 0.02* |
| Limbic striatum | 0.47 ± 1.58 | 0.81 ± 1.31 | T57 = 0.76; P = 0.45 |

Dopamine function is expressed as the mean ± standard deviation z-scores for 18F-DOPA k_i_^cer^ values, representing presynaptic dopamine synthesis capacity.
